# Refining the Mechanism of Drug-Influenced Gingival Enlargement and Its Management

**DOI:** 10.7759/cureus.25009

**Published:** 2022-05-15

**Authors:** Muhammad Annurdin Sabarudin, Haslina Taib, Wan Majdiah Wan Mohamad

**Affiliations:** 1 Department of Periodontology and Community Oral Health, Faculty of Dentistry, Universiti Sains Islam Malaysia, Kuala Lumpur, MYS; 2 Periodontics Unit, School of Dental Sciences, Universiti Sains Malaysia Health Campus, Kelantan, MYS; 3 Clinical Immunology Unit, School of Dental Sciences, Universiti Sains Malaysia Health Campus, Kelantan, MYS

**Keywords:** immunosuppressants, anticonvulsants, calcium channel blockers, drug- influenced gingival enlargement, gingival enlargement

## Abstract

Drug-influenced gingival enlargement (DIGE) or overgrowth manifests as abnormal enlargement of the gingiva due to an adverse effect of certain drug reactions in patients treated with anticonvulsants, immunosuppressants, or calcium channel blockers (CCBs). As the gingival enlargement became significant, it may interfere with the normal oral hygiene measures, aesthetics, as well as masticatory functions of the patients. The exact mechanism of how this undesirable condition develops is yet unknown, and complicated, with non-inflammatory and inflammatory pathways involved. This review illuminates these putative pathways of DIGE and highlights various treatment approaches based on existing research and current observations.

## Introduction and background

Gingival enlargement, initially known as gingival overgrowth, is an enlargement of the gingival tissues that can be localized or generalized. This term has taken the place of gingival hyperplasia (increase in cell number) and gingival hypertrophy (increase in cell size) as these are histological diagnoses and do not fully characterize the pathological processes found within the tissues [1]. It is now understood that true gingival enlargement necessitates changes in the varying cell size, cell multiplication, gingival vasculature, and extracellular matrix to varying degrees [2] which may affects the aesthetic, mastication, speech, and oral hygiene measures [3,4]. Gingival enlargement has been linked to various factors such as adverse drug effects, inflammation, neoplastic processes, and hereditary gingival fibromatosis [5].

Drug-influenced gingival enlargement (DIGE) is an unwanted effect commonly associated with medications [3,4]. Recent study reported that DIGE was prevalent in 77.3% in patients taking antihypertensive medication more than five years [6]. Currently there are twenty prescriptions known to cause DIGE [7]. The most common drug types which have been reported to cause gingival enlargement include antiepileptic drugs such as phenytoin for treatment and control seizure disorders in epileptic patients, antihypertensive agent including calcium channel blockers (CCBs) such as nifedipine and amlodipine, and immunosuppressants such as cyclosporin A to prevent rejection in patients received organ transplants {7}. DIGE may develops in a susceptible individual during the first three months of commencing medications [8].

**Figure 1:**
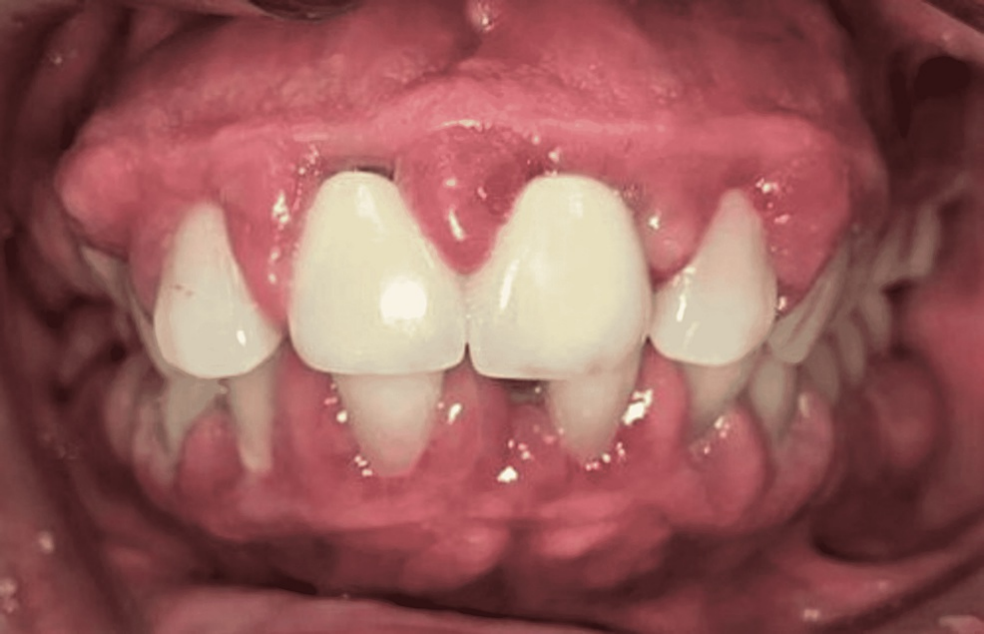
Clinical appearance of drug-influenced gingival enlargement at the anterior dentition in a hypertensive patient who has been on amlodipine 10 mg for three years.

Clinically, DIGE usually affects the interdental gingiva of the anterior teeth and confined to the attached gingiva. It may extend coronally, as the tissue enlarges, become thickened and lobulated appearance (Figure 1). DIGE also has tendency to affect posterior teeth but rarely occurs compared to the anterior region [9]. The enlargement tends to be more pronounced in areas where plaque accumulates, such as at the edges of restorations, retained roots, and around the orthodontic appliances but it seldom seen in edentulous area [10]. The lesion may be inflamed if associated with periodontal infection and appeared as red or purplish in colour, and bleed profusely upon provocation [11].

Histologically, there was an increased in epithelial thickness of the gingiva which commonly observed in nifedipine and cyclosporin A-induced gingival enlargement and is associated with increased mitotic activity, particularly in the spinous layer of oral epithelium [12]. There is an excessive accumulation of extracellular matrix (ECM) protein such as collagen or amorphous ground substance in the gingiva. Besides, various thickness of parakeratinized squamous epithelium with acanthosis covers the connective tissue stroma and elongated rete pegs extending deep into connective tissue, creates irregularly arranged collagen fibers [13]. Another evidence also supports that connective tissue is an important element in the pathophysiology of DIGE as seen by significant increase in connective tissue matrix. An ultrastructural study of DIGE showed that the increase in gingival tissue is based on connective tissue response rather than epithelial cell layer involvement. The lamina propria shows collagen fibrosis, increase of vascularity, infiltration of inflammatory cells containing plasma cells and lymphocytes, and an amorphous ground substance with evident changes of glycosaminoglycans (GAGs) [14,15].

Although DIGE were reported elsewhere, the mechanism underlying this condition remains unclear and thought to be multifactorial [16]. This review aims to highlight further on the pathways involve in the development of DIGE. Besides, the approaches of its management based on the current knowledge are also discussed.

## Review

The mechanism of DIGE

The underlying mechanisms behind DIGE were divided into non-inflammatory (biochemical) and inflammatory pathways [16,17]. The non-inflammatory pathways include 1) inhibitory effect of sodium/calcium ion flux upon cation channels mechanisms, and 2) defective collagenase activity due to decreased uptake of folic acid [16-18]. Meanwhile, the inflammatory pathways include 1) Alteration in the production of inflammatory cytokines and interaction of chemotactic factors and 2) Immunological changes and inflammatory process [5,16,19,20].

Non-inflammatory (biochemical) pathway

Inhibitory Effect of Sodium/Calcium Ion Influx Upon Cation Channels Mechanisms

It has been proposed that all the drugs that induce gingival enlargement, namely anticonvulsants, CCBs, and immunosuppressants have a similar mode of action at the cellular level, by which they inhibit the intracellular calcium (Ca2+) and sodium (Na+) ion influx through cation channels, resulting in decreased folate cellular absorption and consequent folate insufficiency [21,22]. These changes will lead to further effects at the cellular level as explained below.

Defective Collagenase Activity Due to Decreased Uptake of Folic Acid

Folic acid cellular uptake is dependent upon both an active transport regulated channel and passive diffusion [23]. Folate deficiency primarily affects epithelium, gonads, and bone marrow. Since folic acid plays an important role in DNA synthesis, tissues with higher turnover rates like gingival epithelium are often first affected [24]. Decreased folic acid also causes a reduction in the synthesis and activation of certain matrix metalloproteinases (MMPs) which are necessary to convert inactive collagenase to active collagenase within the gingiva. Therefore, an insufficient amount of active collagenase for the breakdown of excess gingival connective tissues results in the development of gingival enlargement [16]. MMPs are a family of more than twenty enzymes which include collagenases, stromelysins, and gelatinases. The imbalance of inhibition and activation of MMPs may result in excessive degradation or accumulation of connective tissues [16].

Additionally, the activation and inhibition of collagenase enzymes is a complex mechanism dependent upon multiple biochemical pathways at the cellular level. The interplay among these biochemical pathways includes transforming growth factor (TGF), tissue inhibitors (TIMP), MMP-1, Smad proteins, E-cadherin, and activator protein (AP)-1 [16]. Smad proteins are intracellular molecules that mediate the intracellular signaling cascade of TGF-β superfamily growth factors. The TGF-β superfamily comprises two groups of growth factors, namely bone morphogenetic proteins (BMPs) and TGF-βs [25]. Ligands of the TGF-β superfamily bind to cell surface receptors to activate Smad proteins in the cytoplasm, then the activated Smad proteins translocate into the nucleus to activate or repress specific target gene transcription. Both groups of growth factors play important roles in regulating a wide range of biological processes such as morphogenesis, embryonic development, adult stem cell differentiation, immune regulation, wound healing, as well as inflammation [25].

Inflammatory pathway

Alteration in the Production of Inflammatory Cytokines and Interaction of Chemotactic Factors

The gingival tissue is subjected to multiple invasions that induce a state of permanent tissue repair involving the inflammatory cells, fibroblasts, and chemotactic factors [26]. For instance, dental plaque biofilm and physical insults contribute to the gingival tissue repair which increases connective tissue build-up by the production of inflammatory cytokines and growth factors such as interleukin-1β (IL-1β), interleukin-6 (IL-6), TGF-β, platelet-derived growth factor (PDGF) and thus, enhance gingival enlargement [26,27]. A synergistic enhancement of collagenous protein synthesis by human gingival fibroblasts was found when these cells were exposed to nifedipine in the inflamed gingival tissues with elevated pro-inflammatory cytokine such as IL-1β [28]. Furthermore, a reported histologic feature of cyclosporin A-influencing gingival enlargement is a dramatic elevation in the expression of IL-6 by the cells within the gingival connective tissues. IL-6 appears to target connective tissue cells such as fibroblasts by enhancing fibroblast proliferation and exerting a positive regulation on collagen as well as GAGs synthesis [29]. Therefore, IL-1β and IL-6 have been proposed to play a pathogenic role in fibrotic diseases such as pulmonary and gingival fibrosis [30,31].

Growth factors (GFs) have been studied as well, and their activation may play an important role in the mechanism of DIGE. Basic fibroblast growth factors are a fibroblast and keratinocyte mitogen molecule with morphogenesis and differentiation functions related to fibroblastic proliferation in gingival enlargement [32]. Vascular endothelial growth factors (VEGF) promote endothelial cell proliferation and differentiation which induces microvascular hyperpermeability and participates in ECM remodeling [33]. Cell migration is an important phenomenon in tissue formation and remodeling that is controlled by epidermal GF. Together with PDGF, it facilitates wound healing and enhances wound strength, by the migration of macrophages and fibroblasts as well as the synthesis of matrix proteins (GAGs, fibronectin, and collagen) [13,34]. In addition, PDGF can stimulate the synthesis of other GFs which are partly mediated by the induction of endogenous GFs such as insulin-like GF (IGF) that causes an increase in fibroblast collagen synthesis [35].

TGF-β1 is an important regulatory cytokine secreted by a variety of cells including macrophages. Apart from being involved in collagen metabolism, it also stimulates the fibroblastic population and the ECM deposit of the fibronectin and GAGs [36]. However, Trackman et al. (2004) stated that TGF-β1 has less magnitude effect in the regulation compared to other connective tissue growth factors. They proposed connective tissue growth factor (CTGF) as a possible matrix stimulatory factor regardless of TGF-β1 in DIGE [19]. On other hand, several studies reported that the binding between CTGF and TGF-β1 reinforces the fibrogenic function of the fibroblast [19,26,37].

In addition, CTGF regulates the proliferation and differentiation of connective tissue cells stimulating ECM production. The binding of CTGF to integrin α6β1 forming an insoluble collagen accumulation was observed to appear in gingival human fibroblast cultures and eventually stimulates ECM production [5]. Trackman et al. (2004) reported that gingiva with more fibrous tissues appears to contain higher levels of CTGF. They had identified clear, consistent molecular, and cellular distinctions among phenytoin, nifedipine, and cyclosporin A-influenced gingival enlargement [19,26].

Besides, fibroblast collagen phagocytosis is the regular catabolism for ECM degradation which is related to some biochemical modifications at the integrin receptors when collagen adheres to the cell membrane. A decrease in the expression of integrin α2 by influencing drugs, or a decrease in collagen adhesion due to the presence of pro-inflammatory cytokines, could inhibit phagocytosis and give rise to the development of gingival enlargement [37-39].

Immunological Changes and Inflammatory Process in DIGE

The immune system consists of two types of defense mechanisms, which are innate immunity and adaptive immunity. Innate immunity is the first line of defense against an invading pathogen; it represents as a barrier function provided by the lining epithelium of different mucosal tissues which contains a number of cells including neutrophils and macrophages, dendritic cells, natural killer cells, and mast cells [40,41]. Meanwhile, adaptive humoral immune defense is antigen-dependent and antigen-specific, thus involving a lag time between exposure to the antigen and maximal response. If innate immunity is ineffective in eliminating infectious agents, adaptive immunity will take place [41].

The immunological changes and inflammatory features associated with DIGE include increased macrophage reparative/proliferative phenotype, up-regulation of essential GF, IL-1β, IL-6, antibodies, and variable lymphocyte proportions [21,42-44]. The inflammatory changes within the tissue may enhance the interaction of calcium and fibroblast cells [45]. As explained previously, the effect of inducing drugs on the sodium-calcium exchange reduces the cationic cell influx intracellularly [46]. Calcium acts as a second messenger by which its regulation depends on mechanisms that control cell membrane flux and its release from intracellular deposits. Calcium binds with proteins and activates target molecules such as enzymes and ionic channels. It also modulates intracellular transcription and proliferation, and functions related to the extracellular matrix through the integrins [47].

Immunoglobulin A (IgA) is the predominant immunoglobulin isotype in human saliva. Salivary IgA has been shown to absorb and affect the adhesion of oral microorganisms in dental plaque biofilm [48]. The resistance of salivary IgA towards proteases makes these antibodies uniquely suited for functioning mucosal secretions [40]. However, in an earlier study, Haldorsen et al. (1977) reported that the concentration of serum IgA decreased in patients receiving long-term phenytoin medication. Patients on phenytoin therapy demonstrated low IgA antibodies which suggest that the drug could be the cause of terminal differentiation failure of IgA-bearing B cells. Although the contribution of anticonvulsant drugs and immunosuppressant drugs to these changes is uncertain, phenytoin has been considered responsible for some specific immune alterations [49]. Furthermore, it was suggested that DIGE affects the mechanisms of the host's immune response, resulting in an increase in gingival mass, and that long-term use of these medications could give rise to a decrease in serum and salivary IgA level, inducing periodontal inflammation. [50].

Prevention and treatment

Patients who are at risk or who have already developed DIGE require close dental care. DIGE management strategies employ a number of treatment approaches which include non-surgical and surgical approaches.

In non-surgical treatment approaches, the modifying factors need to be controlled [51]. Elimination of local factors such as meticulous biofilm control, and regular periodontal maintenance is essential to prevent the recurrence of DIGE. The presence of retentive factors, either associated with the tooth anatomy or more frequently, due to improper restorative margins, are often associated with gingival inflammation and/or clinical attachment loss, that should be eliminated to reduce the impact on periodontal health. Orthodontic bands and/or appliances at the region of gingival enlargement should be removed. Professional mechanical plaque removal including scaling and root surface debridement has been shown to offer some relief in DIGE patients [52]. In addition, the use of systemic antibiotics, such as a short course of azithromycin (3-5 days, 250 to 500 mg/day) may affect DIGE remission which ranges between three months to two years [53]. However, another study found that a seven-day course of azithromycin (or metronidazole) does not improve remission of DIGE, although it acts on concomitant bacterial over the infection and gingival inflammation [54]. It can be concluded that there was insufficient evidence for systemic antimicrobials as an efficacious therapy for DIGE and future studies are necessary [16].

With regards to DIGE by CCBs, a multidisciplinary input or onward referral to the physician may be required in severe DIGE cases (for withdrawal, or substitution of medication). When this treatment approach is taken, it may take one to eight weeks for the remission of DIGE [55]. However, the response to this treatment approach seems unpredictable, especially in those with long-standing DIGE [56]. The most effective treatment of these lesions is the cessation of the offending medication and substitution with another class, or a cocktail, of antihypertensive drugs by the physician. These include B-blockers, diuretics, or angiotensin-converting enzyme inhibitors as DIGE has not been reported with any of these drugs [57]. Another option is substitution with another CCB drug that has a lower risk of inducing gingival enlargement such as verapamil or isradipine [58,59]. If regimen change is not an option, the lesions should be managed with or without surgical intervention.

However, when the growth compromises oral hygiene procedures or chewing function, as well as aesthetics, surgical removal of DIGE is usually recommended. For this approach, the gingivectomy and/or gingivoplasty by internal/external bevel incision could be done. Few techniques for surgical interventions can be applied such as scalpel gingivectomy, electrosurgery, and laser approaches. Occasionally, it may be technically sensitive or impractical for example in pediatrics and special needs children, or in patients suffering from impaired hemostasis [59]. In these situations, the use of electrosurgery or laser would be more recommended. The use of lasers has shown predictive outcome and produces good hemostasis with less pain during the procedure and post operatively as well [60,61].

## Conclusions

To date, the mechanism of DIGE development is complex in that it is applicable to all common drugs affecting gingival enlargement. Further molecular approaches are needed to clearly establish the mechanism of DIGE and to provide better therapeutic modalities.
